# Don’t forget the boundary problem! How EM field topology can address the overlooked cousin to the binding problem for consciousness

**DOI:** 10.3389/fnhum.2023.1233119

**Published:** 2023-08-03

**Authors:** Andrés Gómez-Emilsson, Chris Percy

**Affiliations:** ^1^Qualia Research Institute, San Francisco, CA, United States; ^2^College of Arts, Humanities and Education, University of Derby, Derby, United Kingdom

**Keywords:** consciousness, binding problem, combination problem, boundary problem, electromagnetic fields

## Abstract

The boundary problem is related to the binding problem, part of a family of puzzles and phenomenal experiences that theories of consciousness (ToC) must either explain or eliminate. By comparison with the phenomenal binding problem, the boundary problem has received very little scholarly attention since first framed in detail by Rosenberg in 1998, despite discussion by Chalmers in his widely cited 2016 work on the combination problem. However, any ToC that addresses the binding problem must also address the boundary problem. The binding problem asks how a unified first person perspective (1PP) can bind experiences across multiple physically distinct activities, whether billions of individual neurons firing or some other underlying phenomenon. To a first approximation, the boundary problem asks why we experience hard boundaries around those unified 1PPs and why the boundaries operate at their apparent spatiotemporal scale. We review recent discussion of the boundary problem, identifying several promising avenues but none that yet address all aspects of the problem. We set out five specific boundary problems to aid precision in future efforts. We also examine electromagnetic (EM) field theories in detail, given their previous success with the binding problem, and introduce a feature with the necessary characteristics to address the boundary problem at a conceptual level. Topological segmentation can, in principle, create exactly the hard boundaries desired, enclosing holistic, frame-invariant units capable of effecting downward causality. The conclusion outlines a programme for testing this concept, describing how it might also differentiate between competing EM ToCs.

## 1. Introduction

This paper elucidates a key challenge for theories of consciousness, the boundary problem, explaining its historical context, how it has not yet been adequately addressed, and a potential solution for electromagnetic theories based on topological segmentation.

Using the Seth and Bayne (2022) taxonomy, this paper focuses on the set of theories of consciousness (ToCs) that attempt to explain why some systems have subjective awareness with a particular phenomenology (Nagel, 1974) and others do not; specifically, what is the mechanism that results in a first person perspective (1PP).

We trust that the notion of a 1PP is understood by readers, as a version of it is near-universally experienced by awake, healthy humans. However, we emphasise that the term is used in an inclusive and basic sense: it corresponds solely to the locus at which the experience arises, rather than some more sophisticated experience of a particular singular self or self-awareness. In other words, the 1PP is consistent with phenomenal experiences of no-self, universal oneness, or fractured/multiple selves.^1^ Similarly, we are focussed on the container of consciousness, rather than its contents; the hard problem rather than the easy problem (Chalmers, 1995); and unified experience as related to the global phenomenal binding problem rather than local, feature-specific, or computational binding (Revonsuo, 1999; Garson, 2001).

Our 1PP has a number of features which any prospective ToC must address. Winters (2021) states that our consciousness often appears unified and compositional; its contents are specific and meaningful, existing from a subjective point of view; it is temporally continuous and limited but coherent. Bayne (2010) provides a detailed account of how our consciousness is unified, typically integrating multiple parts. In other words, our consciousness somehow binds multiple discrete features into a single unified awareness (the binding problem). At the same time, our consciousness does not “bind” features without limit—what we experience varies over time and is thus always strictly a subset of what could be experienced. There is an edge to our awareness, a boundary around us that is generally felt to exist at the human-scale of experience, rather than at the cellular or societal level. While the unity of consciousness and the binding problem have received significant treatment, this article argues that the boundary problem has been inadequately addressed.

The boundary problem is not a trivial matter: any ToC that addresses the binding problem must also address the boundary problem. To a first approximation: once you’ve proposed a binding mechanism that creates larger, unified, macro 1PPs, what mechanism puts a stop to that process? What causes ontologically hard boundaries around these 1PPs and why do the boundaries appear where they do? If the binding mechanism could apply in principle to units smaller and larger than the human brain, what is happening in these cases? Why is our 1PP consistently at the meso-scale of human experience?

We begin with a literature review of the boundary problem, arguing that only a handful of papers have addressed it directly, while several others have groped toward the issue but struggled to capture it fully. None of them have yet provided a full account of all aspects of the explanatory challenge. In the third section, we refine the boundary problem issue into five specific problems, to aid assessments of which ones any given account might successfully address. In the fourth section, we present a conceptual overview of a promising novel avenue for addressing the problem, building on the physical features of topologically segmented electromagnetic (EM) fields and the potential for such a mechanism to generate hard boundaries. By placing a focus on topological segmentation, we tie together other desiderata in a ToC, notably: non-epiphenomenalism, no strong emergence, and frame invariance. Having established topologically segmented pockets as a conceptually sound mechanism for addressing the boundary problem, we use the conclusion to outline a simulation and empirical research programme for testing this concept. Such an exercise would also help differentiate between competing EM ToCs by analysing the different spatial scales at which they locate moments of experience.

## 2. Literature review of the boundary problem

In this section, we establish Rosenberg as the leading scholar to specify the boundary problem in detail, acknowledging others having touched on the issue previously. A systematic literature review using the Scopus dataset and citation tracing allows us to describe recent attempts to address the boundary problem, whether by name or by indirect discussion of its issues, before refining it into five specific problems in section “3. Precise statement of five specific boundary problems.” Finally in this section, we review a range of EM ToCs to explore how they might address the boundary problem, whether explicitly or implicitly, before introducing in section “4. Topological segmentation of EM fields as a resolution direction” as aspect of EM field that has the characteristics to defeat the boundary problem but has not yet been capitalised on as such in EM ToCs.

### 2.1. Rosenberg’s formulation of the boundary problem

Rosenberg (1998) is credited by Chalmers (2016) as the originator of the boundary problem concept, being one of several combination problem issues that Chalmers raises for theories of panpsychism.

Later authors have correctly flagged that these combination issues often apply to other ToCs as well, at least insofar as they wish to explain in a physically grounded fashion the complex, unified, bound macrophenomenology that humans typically experience (Mendelovici, 2019). Indeed Johnson (2016) adopts the same terminology in *Principia Qualia*, in which the boundary problem is one of eight subproblems of consciousness that all ToCs need to address: “how to determine the correct boundaries of a conscious system in a principled way.”

For instance, information or causality driven solutions to the binding problem, e.g., functional or computational theories of mind (discussion in Gómez-Emilsson and Percy, 2022), might define phenomenal binding as occurring when two items interact causally or are associated with each other in a database. The challenge is that there is no neat boundary where the causal interactions or informational associations should stop—the solution over-delivers and everything ends up bound together. It would be necessary to define subtypes of causality or information linkage which generate phenomenal binding while others do not.

Such subtype definitions need to address topologies that are likely to arise in complex real world environments. For instance, it may be possible to define multiple, separate local maxima of connectivity (or high points of any mathematical construct used in the ToC) as differentiated from their surroundings by moats of low connectivity areas. However, in a continually changing, interacting environmental topology of connectivity strengths, it is necessary to address the challenge of separating systems from subsystems in a disciplined manner, particularly if a hard boundary is desired. How much lower the moat needs to be than the local maximum risks becoming an arbitrary distinction. Further, if a local maximum of connectivity is to have sufficient complexity to correspond to a 1PP that captures all the different experiences we typically have in a single moment, then it is likely to have its own internally varied topology. Such variations would likely create internal moats of relatively lower connectivity and yet more local maxima—how are these to be treated in the theory?

The requirements for such definitions include that subtypes or thresholds are not arbitrary, that the differentiations have a mechanic motivation for the presence/absence of binding, and that the definition encompasses binding such as it occurs in the human system. This issue was pointed out by Rosenberg and discussed indirectly by Bell (2005:165), e.g., “the wheel regarded as a wheel is discrete, but regarded as a piece of matter, it is continuous.” The discreteness of the first perspective likely requires a third party’s instrumental perspective, such as a human observer looking for something that would roll across the ground. Other third party observers may not recognise that first perspective, perhaps ants climbing over the wheel seeing it as a mountain growing out of the ground or hypothetical alien entities perceiving the world via radiowaves who scarcely see it at all. The second perspective of continuity is a more plausibly neutral, basal view that would be consistent across all observers and none.

Broadcasting to a global brainwide system or workspace might also be taken as the axiomatic definition of 1PP, e.g., Dehaene (2014), although Baars (1997) did not support this approach and was criticised as such for not addressing the hard problem (Dalton, 1997). In one sense, this resolves the boundary problem, by defining it axiomatically at the edges of the human brain. However, this means the ToC by definition either declares all non-human systems lacking a 1PP (including possible future evolutions of the human brain)^2^ or is unable to comment on them and is therefore not a general ToC. In another sense, we might associate the 1PP with a general, substrate neutral workspace, which would lead back to the challenges in the previous paragraph for computationalists.

Rosenberg (2004, 2014) develops the boundary problem concepts further in his 2004 and 2014 accounts, framed in a general way that do not apply uniquely to one subtype of ToCs. Chalmers summarises Rosenberg’s argument as “how do microexperiences come together to yield a bounded consciousness” but does not treat it as one of the three main aspects of the combination problem in his 2016 discussion. However, reviewing Rosenberg’s full development of the boundary problem, there are novel issues raised which recent discussions of the unity of consciousness problem and the binding problem^3^ grasp at but do not fully capture.

Rosenberg’s original question concerns “how consciousness can exist at the middle level of nature” (2004, s4.1). He explains how the human body, as with other objects we observe at the middle level of nature, has only an intuitive, specious solidity within a particular boundary. Where you draw the boundaries depends on a particular observer’s perspective and interests. In his words, “a cell may be an individual; also, at the same time, it may be part of an organ; at the same time, it may be part of an individuated bodily system such as the reproductive system; at the same time, it may be part of the organism as a whole and part of that organism’s society; at the same time, it might be part of an ecosystem” (2004, s4.2).

To help us realise the arbitrariness of our strong intuitions of solidity, Rosenberg points to various psychiatric phenomena, thought experiments with human relay mechanisms in giant systems isomorphic to fish brains, and a vivid account of the complexity of the US economy. Rosenberg describes our account of the familiar middle layer of experience as trapped between a Scylla and a Charybdis, where the Scylla refers to why our experience does not exist at the subsystem level, i.e., the Russian dolls nested within us, and the Charybdis asks the parallel question of the larger systems within which individual human entities are nested.

Various mechanisms proposed for phenomenal binding establish in principle why the Scylla can be avoided. However, why does the merging stop at the meso-level of human experience? If researchers cannot define a mechanism that creates a hard boundary at the level of the brain (or their chosen space corresponding to our meso-level unified experience) but soft boundaries at every lower level, they must accept that merging into higher levels would happen under certain circumstances.

Rosenberg is not the first to identify this problem, although his is the earliest detailed treatment we have identified. Nobel-prize winning Schrödinger (1951) agonised over the same question, drawing on similar observations as Rosenberg in a brief paragraph discussion in his 1951 book: “Why is it precisely at this *intermediate level* in the hierarchy of successively superimposed unities (cell, organ, human body, state)—why, I ask, it is precisely at the level of my body that unitary self-consciousness comes into the picture, whereas the cell and the organ do not as yet possess it and the state possesses it no longer?” (p. 33, emphasis in original). Schrödinger at least did not doubt the difficulty of this question, indeed it was part of his “impenetrable thicket of questions” (p. 33) that arrived when he thought about his introspected unity of the self.

In section “3. Precise statement of five specific boundary problems,” we unpack Rosenberg’s account in more detail and take inspiration from it to specify five specific problems that help set out the full range of challenges for a candidate ToC to address.

### 2.2. Literature review of the boundary problem

Despite identified above as a distinct problem for ToCs to address, the boundary problem has received little scholarly attention, with only a few papers that address the topic directly and a few that address it indirectly.

Searches in the Scopus database in March 2023 identified 92 papers with the “binding problem” in the title, abstract or key words, requiring also at least one of the following to increase the probability of topic relevance: consciousness, conscious, qualia, “philosophy of mind.” A total of 47 were identified for the “combination problem.” Only 5 were identified for the “boundary problem,” of which three are relevant for discussion below.^4^ Citation tracing in Google Scholar of Rosenberg’s three papers referenced in section “2.1. Rosenberg’s formulation of the boundary problem” did not identify any further researchers proposing features of ToCs that would resolve his concerns.

Fekete et al. (2016) discuss a similar “boundary problem,” albeit restricted to computational ToCs. They report the idea as original but without citing Rosenberg’s work. In their presentation, the relevant boundary problem is that if quantitative, graded measures of consciousness label a particular system as conscious, it would often also label some of its subsystems and irrelevantly extended systems as conscious. The described issue is that this either leads to a bizarre proliferation of minds or to the possibility of various appendages/subsystems/extensions labelled as conscious that are epiphenomenal to the main system measure of consciousness.

Fekete et al. (2016) suggest a solution, asking researchers to look for properties that offer a principled mechanism for singling out intrinsic systems, demarcating systems as a matter of fact rather than being a matter of interpretation from different observers’ viewpoints. Later discussions in this section on phase transitions and the field topology in section “4. Topological segmentation of EM fields as a resolution direction” are suggested as examples of such intrinsic mechanisms. We build on Fekete et al. by specifying a broader set of specific problems and extending the analysis to any ToC that claims to solve the binding problem, being an issue for most non-mysterian ToCs grounded in physical reality, whether via supervenient, weakly emergent, or implementational relationships.

The other two papers from the Scopus review are from Hunt, of which the first, Hunt (2016) criticises IIT’s approach to the boundary problem issues, i.e., the exclusion principle by which only the subsystem with the most integrated information (highest phi) possesses phenomenal consciousness out of all those available within a given system. IIT remains under development with recent theoretical overviews available in Oizumi et al. (2014) and Barbosa et al. (2021). In brief, IIT equates a system’s consciousness to its causal properties, which can be measured mathematically (“phi”) via algorithms mapped from five specific properties claimed to capture human phenomenology. Causal integration of a particular type is proposed as the binding mechanism.

In later work, Hunt (2020) discusses how the slowest shared resonance could motivate boundaries in the general resonance theory of consciousness (GRT), providing a detailed mathematical heuristic for calculating the specific spatiotemporal boundaries of any conscious entity. GRT similarly remains in development, with its main theoretical approach described in Hunt and Schooler (2019) and Hunt (2020). In brief, GRT starts from panpsychism and explains how shared resonances between micro-consciousness entities can overcome the binding problem. The slowest shared resonance across different systems is taken to define the subsequent scope of macro-consciousness, with electromagnetic field synchrony (resonance) being the primary type of shared resonance relevant to the scale of human life and consciousness. In other words, GRT is primarily an EM field theory of consciousness in terms of its practical implications.

We welcome these researchers’ work on this topic and see these as potential avenues that could be further explored to address Rosenberg’s concerns more fully. For instance, with Rosenberg’s Charybdis, the current computational difficulty of calculating phi for real-world systems (e.g., Kim et al., 2018) limits confidence in identifying candidate system and subsystem boundaries. Such analyses would be needed to test the hard boundary that is supposed to exclude the cerebellum from the cerebral cortex 1PP or the soft boundaries between subsystems within the cerebral cortex that are to be extinguished by the exclusion principle (Tononi, 2015). Further, if the cerebral cortex is damaged and later repaired, why does our 1PP (appear to) disappear rather than shifting for the interim to the next highest phi subsystem in the overall system, e.g., a cerebellum module or elsewhere?

GRT also needs to address even slower shared resonances in larger interpersonal systems, such as singing choirs or rhythmic dancing. Indeed some have argued that GRT may therefore be able to account for phenomena such as group consciousness (Young et al., 2022). It is possible that all nested levels of resonance represent separate levels of consciousness, as Hunt (2020) suggests, but then we must explain why the human experience is so consistently at the meso level. Alternatively, there may be some phase transition mechanics, pointed to by Hunt and Schooler (2019), that means such macro-level phenomena beyond a single human entity fail to establish resonance of a level to drive consciousness. One such heuristic is proposed in Hunt (2020) – using various candidate synchronicity indexes to measure complex resonance chains – paving the way for further mathematical and empirical work to test it.

### 2.3. Indirect discussions of the problem

In addition to the structured database search and citation tracing above, we selectively reviewed other studies that might indirectly address the boundary problem.

Bayne (2010) discusses the binding problem at length, but does not address the boundary issues, with no citation of Rosenberg and no explicit discussion of the boundary problem. An indirect discussion suggests an assumed possible resolution: “the biological account enables us to determine the boundaries between selves with relative ease,” i.e., presumably boundaries of the physical body/brain system. However, there is no further discussion that would account for the challenges Rosenberg raises. In his 2014 response to reviewers’ comments, Bayne (2014) surfaces his difficulty with the underlying issue, albeit without the precision that comes with Rosenberg’s framing of it. Bayne suggests the reviewer is asking for “an account of why particular experiences are parts of the experiences that they are parts of whereas others are not,” which Rosenberg might translate into why some experiences are contained within a given phenomenal boundary whereas others are not. Bayne states that he is unsure what it would take to answer such a question and would welcome a theory that could do this as part of a comprehensive ToC, but is not himself advancing one at this stage. Goff (2020) highlights the combination problem as an important open problem for panpsychism, with several promising avenues, but does not draw out the related boundary problem issues or cite Rosenberg.

Winters (2021) also gets close to the boundary problem in his comparison of how different ToCs account for the “limited and coherent” nature of our phenomenality, although his primary focus is on why the majority of incoming data streams go unnoticed by our 1PP, i.e., unconscious information processing. Nonetheless, his broader discussions of this point contain explanation that address aspects of Rosenberg’s concern. His preferred theory looks at systems of temporally integrated causality (TIC), arguing that the system containing the highest degree of TIC is naturally bound by surrounding areas of lower TIC. While this avenue may be vulnerable to the same concern as with IIT and other computational approaches specified above, it nonetheless has the potential in principle to address important aspects of the boundary problem.

Bond (2023) suggests coherence fields as atomic nodes within expanses of integrating photonic waves as the fundamental units of 1PP. While part of his discussion captures a “fading with distance” logic, it is also possible that a phase transition between coherence/decoherence in his electric currents might motivate a hard boundary. Keppler (2021) similarly appeals to phase transitions, pointing to superradiance as a potential model to follow. In both cases, the details by which the mechanism would produce an ontologically hard boundary are not fully explicated, with the underlying physics perhaps yet to be fully understood. Nonetheless, as with resonance theories, such potential phase transition mechanisms are a fruitful avenue for future research. A more directly specified phase transition is the pre-collapse quantum entanglement motivated as the basis for consciousness by Barkai (2018), subject to ongoing research into the maintenance of entanglement at sufficient scales to motivate the complexity and diversity of our phenomenal experience (e.g., Goh et al., 2020).

In general, further mathematical and empirical work in these areas, as with GRT, IIT, TIC, or Fekete’s system properties, may lead to more precisely specified phase transitions and boundary problem solutions. Their mathematical properties can then be tested against boundary identification in topologically complex environments (like the example in section “2.1. Rosenberg’s formulation of the boundary problem”) and the five boundary problems in this paper, alongside other requirements for a ToC. These avenues all merit exploring as potential solutions.

### 2.4. Further EM-field theory perspectives on the boundary problem

Finally, we investigate additional high profile EM theories to assess their explicit or implicit positions on the issues posed by the boundary problem, focussing on EM field theories given their success with the binding problem and because the conceptual solution we propose in section “4. Topological segmentation of EM fields as a resolution direction” is a tool available to such field theories.

EM-field ToCs regularly reference the ease with which they defeat the binding problem, typically noting that fields are ontologically unified by their physical nature and automatically integrate all the information contained in the underlying EM activity that generates them (Jones, 2016; Keppler, 2021; Ward and Guevara, 2022; McFadden, 2023; etc.). However, the discussion of boundary problem issues is typically more implicit and does not account for all the concerns raised or implied by Rosenberg. In these additional EM ToC papers reviewed, we did not identify any to address the problem by name, cite Rosenberg, or discuss the full extent of the potential explanatory challenge.

In Jones (2010, 2019) “realist field theory” it is the energy in EM fields that is conscious, unlike for instance the way consciousness emerges out of integrated information in McFadden’s ToC, provided those fields also have influence over motor activity to avoid the pointlessness of epiphenomenality. Jones sees all such fields as conscious, but it is only when they bind together into larger EM fields that we get the kind of complex macrosubject consciousness of primary interest (i.e., our own first person perspectives). This suggests a continuous increase in scale rather than something with a phase transition demarcating consciousness being on or off.

A closer discussion of boundary problem issues comes in Jones (2013) discussion of how EM fields can be consistent with mental privacy, e.g., no telepathy even when our brains are close together such that some of the EM fields might overlap or merge. His solution is to identify consciousness of the relevant scale only in highly localised fields, unlike for instance the larger, more brain-wide fields of McFadden’s ToC. By requiring more local, stronger fields created in ion currents, the rapid decline in EM field strength with distance is sufficient that nothing gets past the boundaries of the physical brain and thus into telepathic territory. This perhaps resolves the “macro” side of Rosenberg’s problem, but not the “micro” side. There must be many candidates for boundaries within the brain that enclose sufficiently strong EM fields; why does it appear—at least most of the time—that we only experience one? Which one is it and why?

Invocations of rapid declining strength with distance are found elsewhere in EM-theorists’ accounts of boundaries. Ward and Guevara (2022) say at “distances larger than a few centimetres, the activity of non-synchronised neuronal networks is indistinguishable from neural noise.” However, Ward and Guevara acknowledge the deeper issues of Rosenberg’s concern, noting that the brain has many EM fields, with various nested and overlapping sizes and boundaries, and asking which create our subjective perspectives.

Ward and Guevara (2022) set out the case for a particular EM field generated by the thalamus, suggesting that axiomatically a 1PP is an EM field that expresses a model of an external environment, a self-entity, and various actions that entity can take to influence its environment. Other fields do not express such models, although they may interface with it (e.g., subconscious inputs into our 1PP), and hence are not themselves phenomenally conscious—they lack a 1PP. This is a powerful argument, although it presumably implies that even relatively simple EM systems (which cannot but generate EM fields) would naturally have fields endowed with 1PP. For instance, a digital computer using a camera, a robotic hand, and AlphaGo to play a game of Go would meet a de minimus version of these requirements. If this is inadequately “complex” or “intense” in some manner to spark a true 1PP, then we are back to a fuzzy boundary or some unspecified phase transition.

Another field related theory invokes phase transitions, this time in the zero point field (ZPF), the ubiquitous substrate understood to mediate the EM force in quantum electrodynamics (Keppler, 2021). Shani and Keppler (2018) present an explanation of how cosmo-panpsychism can address various (de)combination problems, including a discussion of boundary formation, carving out smaller 1PP entities out of the omnipresent zero-point field underlying all quantum behaviours. They describe a “clear demarcation criterion between conscious and non-conscious systems in such a way that the formation of transiently stable attractors distinguishing themselves by a high degree of coherence is an essential prerequisite for conscious processes.” From one perspective, they describe a continuous spectrum of increasing consciousness: “simple quantum systems, such as atoms and molecules, are probably equipped with a very rudimentary, limited, and monotonous form of consciousness.” From another, they suggest possible phase transitions that might point to strong boundaries, whereby “system-specific ZPF modes undergo a phase-locked coupling (accompanying the formation of an attractor) while all the other modes remain unaffected.” Various intensified regions are described as vortices “in constant interaction with, yet functionally distinct from, the surrounding field.”

McFadden invokes a metaphysical argument to explain mental privacy as discussed by Jones (2013). The information in fields, when experienced privately from the inside, has a 1PP phenomenology. From the outside, the same information can be read but not experienced. Such principles can be applied outside of fields as well, as in the relativistic theory of consciousness from Lahav and Neemeh (2022) which can be applied in any physicalist setting. However, in the specific EM setting, we can ask what happens when EM fields from one brain merge with EM fields from another brain. Elsewhere McFadden (2006) says that the high conductivity of the cerebral fluid creates an effective Faraday cage, such that external fields would not influence what happens on the inside. On this account, if the fluid were somehow safely removed or some functionality used to connect fields between brains, we would expect a single merged 1PP emerging from the two 1PPs in two adjacent human brains. McFadden (2020) later invokes an unspecified threshold argument which might assume away the practical feasibility of such merging: “minimal characteristic of an EM field to qualify as conscious must surely be that it possesses sufficient complexity.”

## 3. Precise statement of five specific boundary problems

The original formulations and reformulations of the boundary problem (Rosenberg, 1998; Fekete et al., 2016; Johnson, 2016) can be usefully translated into five specific problems, briefly summarised below before discussing each in detail. The gauntlet for candidate ToCs is to identify the resolution (eliminative or otherwise) they favour, explaining the mechanism that drives it and its consistency with the rest of their theory.

• **The hard boundary problem**. We experience, at least sometimes, an absolute boundary between our 1PP and the outside world, which consists of events that could be experienced by us and are currently being experienced by others. What produces this absolute, i.e., non-fuzzy boundary?
• **The lower-levels boundary problem**. Our 1PP is capable of experiencing diverse and multiple experiences, often simultaneously or co-jointly. To the extent our 1PP is considered to arise out of a complex, multi-part mechanism, there could be boundaries, whether hard or fuzzy, that enclose some of these nested or constitutive sub-experiences. Are there 1PPs also existing at these lower levels? If not, what creates the important boundary at our meso-level but over-rides or renders meaningless any lower level boundaries? If yes, what mechanism ensures our 1PP remains at the meso level of human experience and not also at some of those lower levels, at least at times?
• **The higher-levels boundary problem.** Our 1PP is in turn nested in larger more complex structures. At times, these operate with considerable physical and causal synchrony, such as singing choirs or football teams. Other times, our collective behaviour has resulted in complex systems, often with a degree of persistence through time and emergent properties, such as cities, price setting and goods transfer via marketplaces, and large language models. Are there 1PPs also existing at these higher levels? If not, what makes the meso-level boundary qualitatively different to each higher level boundary? If yes, what mechanism ensures our 1PP remains at the meso level of human experience and not also at some of those higher levels, at least at times?
• **The private boundary problem.** The boundary we experience, whether hard or fuzzy, demarcates our phenomenal experiences from the external environment, which includes other people’s first person perspectives, i.e., other meso entities separate from our own as well as other physical phenomena more generally. What mechanism makes this a private boundary? Why can we not routinely bridge into others’ minds?
• **The temporal boundary problem.** There are often multiple possible bounded entities in a given system, potentially with many at similar, lower, and higher spatial levels. These entities might persist, for some definition of pattern stability, for different, overlapping periods of time. However, our normal experience is typically of a single bounded entity persisting through time, at least for some non-trivial periods of time. How is a broadly stable sense of self stitched together across a sequence of such entities?

### 3.1. The hard boundary problem

The hard boundary problem is one of the introspective explananda of consciousness. It observes that our phenomenal field, or our first person perspective, appears to be enclosed by a firm, absolute boundary. There is a qualitative difference between the things that enter that phenomenal field and things that do not. Such an approach does not deny highly varied experiences within that boundary, potentially including gradations of how “aware” or how “conscious” (in the vernacular sense) we sometimes feel. Drifting in and out of sleep, fading into anaesthesia—such experiences still happen within a boundary, they merely refer to the contents within that boundary gradually dissipating.

The strong statement of this explanandum is that our first person perspective is always walled by a hard boundary. The weak statement is that our first person perspective is at least sometimes walled by a hard boundary. For our purposes, the weak statement is sufficient to demand some explanation of how the perceived hard boundary might be generated. As with all introspective explananda, the hard boundary problem takes its force either from a reader’s ability to relate to it in their own introspection or, in its weak statement, a willingness to believe or an ability to empathise with the self-report of others who claim to have experienced it, as we do.

At least two eliminative responses can be made to the hard boundary problem. One might argue back from introspection to assert that if we probe the very edges of our awareness, the boundaries are in fact fuzzy rather than hard, although arguably this would leave the weak statement intact. For instance, Lidström and Allen (2021) suggest there are no clear boundaries between conscious and unconscious behaviour, so perhaps there is similarly no true boundary in its phenomenology. This account appears to be adopted by some EM-field theorists, as discussed in section “2. Literature review of the boundary problem,” motivated perhaps by the very rapid decline in EM field strength with distance which creates a “sufficiently” hard boundary to account for the introspective phenomenality.

Another eliminative account might argue from indirect realism that when we perceive a hard boundary, this is only an illusion. The underlying reality is continuous, but the complex processing of our perceptual apparatus sees fit to present it as firmly bounded. In this case, the processing mechanism needs accounting for as does the evolutionary argument for why our brain has found reproductive fitness in that particular simplification of presentation. More subtly, such illusory claims only move the explanandum rather than remove it. The very illusion of unity *as an experience* is itself something that requires multiple pieces of information to be simultaneously expressed and phenomenally bound—surfacing a hard boundary indirectly.

If these eliminative responses are not palatable, then some mechanism needs to be explained by which nature produces a hard boundary. Indeed, the problem gains its force because most phenomena that might be co-opted to explain consciousness, at least at the meso level of human experience, appear to be continuous in nature. Many events are synchronous with those that appear in our first person perspective, but do not themselves appear in that perspective. At the end of any edge of physical causality, information-exchange, or spatial proximity in a particular system are yet more proximate interactions, whether extending out forever or nested internally. This includes interactions that have complex feedback loops beyond an immediate biological unit (e.g., for the body when typing on a keyboard or driving a car, or the gut nervous system as connected to the brain stem). EM fields do not automatically close in neat boundaries, they extend out forever, albeit weakening rapidly with the cube of distance.

To fully resolve the hard boundary problem, we wish to identify a mechanism that results in an ontological boundary that is impermeable to the relevant processes, patterns, or substances determined to make up the 1PP.

One example given in section “2. Literature review of the boundary problem” is when systems are in exact resonance with each other, provided we do not need any appeal to resonances lower or higher. A more general phenomenon to call upon is that of phase transitions, although a full solution must identify where the transition takes place, the mechanism that drives it, and motivate why the hard boundary it generates is adequate for enclosing phenomenal consciousness.

### 3.2. The lower-levels boundary problem

The lower- and higher-levels problems correspond to Rosenberg’s original question of “how consciousness can exist at the *middle* level of nature” (2004, s4.1, emphasis added). Appealing to visually apparent “physical boundaries” is not as successful as it might first seem. An alien observer whose eyes exist at the pixel scale of micrometres or light-years would see the physical boundaries of our seemingly solid human system very differently, porous in the former and imperceptibly blended in the latter. Even more strongly, consider an alien observer who does not rely on photons to construct a model of the outside world, but instead relies solely on senses of sound waves or gravitational waves. The boundaries of the human system relative to the lower/higher levels around it no longer necessarily look as unique.

One response to this challenge, common to panpsychic arguments, is to accept (indeed, to celebrate) that all of these different levels have appropriately many 1PPs of differing complexity and persistence. Advocates would ask how we can be sure that the other levels do not exist, given there is no reason to think evolution had to equip us with the senses to perceive them or communicate with them. Is the notion really more bizarre than black holes and the dual slit experiment in physics, or split-brain experiences and Cotard syndrome in neuropsychology? Our intuitions often fail us when extrapolated beyond our comfortable, common surroundings. However, even if such a pluralising account were advanced, we must have some explanation for why our experience is consistently at the meso-level.

Any other response must meet Rosenberg’s challenge—we must identify a mechanism that pushes the 1PP out from the subatomic level but only just so, only up to the human level and no further. This is primarily a question for the natural sciences: boundary-making mechanisms must be identified and tested, both for their ability to generate the necessary features in principle and for whether they actually operate in that way in the human system.

### 3.3. The higher-levels boundary problem

Having explained the lower-levels problem, the higher-levels problem can be understood as the symmetrical question applied to entities larger than the human brain within which the brain is nested. However, it is worth illustrating as a distinct problem because some ToC mechanisms address one but not the other.

Rosenberg (2004) explains how Lockwood’s materialism might solve the lower-levels problem by binding together small experiences via the lines of interactions in the world, but it does not explain why it stops at the human level, given we also nest within larger systems of interactions. Microexperiential panpsychists that do not incorporate binding mechanisms do not have to worry about the higher-levels problem, but struggle to explain why any experience exists at our meso-level at all, perhaps needing to motivate the apparent multi-feature complexity of our experience as illusory.

### 3.4. The private boundary problem

Jones (2013) discusses the experience of mental privacy as one that ToCs should account for. This is a distinct problem from the lower-levels and higher-levels problems, since the latter are focussed on the subsystems nested within us or the macrosystems we nest within, whereas the private boundary problem is between meso-level entities. For instance, if the coherence of consciousness is laid at the foot of neural synchrony, resonant frequencies, or EM fields, then why does our consciousness not somehow link with or merge into another person’s consciousness when they are sufficiently in sync, in resonance, or connected via EM fields with us?

Several EM fields have a natural privacy-preserving explanation for this part of the boundary problem and some resonance theorists explicitly allow for privacy to be violated, as discussed in the literature review in section two. Neuralink advocates would likely argue this is a technical barrier to overcome, rather than an absolute philosophical or biological barrier. Some experiments point weakly to this barrier being weakened today and some studies on conjoined twin suggest that the privacy of a single brain is not absolute (de Haan et al., 2020).

Any of these accounts are adequate in principle for resolving the private boundary problem. The duty on ToC theorists is to choose their preferred explanation and explain why it is consistent with the other binding and boundary problems and with their broader vision of consciousness and human experience.

### 3.5. The temporal boundary problem

The binding problem and associated boundary problems typically begin with a static view of features and experiences. The feature binding problem asks how our experience of a blue chair knits together all the individual neural signals corresponding to bits of the chair into an overall shape and colour.^5^ The phenomenal binding problem asks how all the different experiences in a moment can be experienced from a single unified perspective: perhaps the chair is experienced in a garden along with a particular smell and an unrelated twinge of back pain. Once some mechanism for phenomenal binding is specified, the boundary problem interrogates why the mechanism stops where it does and the consistency of such explanations.

For a static moment, the concept of bound experiences within a boundary can be well defined. For a subsequent moment, the experiences being bound together will often be different and the boundary of 1PP may have shifted. As such, additional constraints are imposed by the requirement to line up with the common, awake and sober experience of continuous perception from one moment to the next. The boundary of our 1PP may sometimes feel like it is contracting or expanding, but it will generally do so without severe discontinuities. The temporal binding problem asks how the moments are knitted together over time to feel like part of the same experience. The temporal boundary problem asks how, once we have a boundary around a static experience or a particular moment of 1PP, that boundary can shift mostly contiguously to have different shapes in future moments. In both cases, we require an explanation that allows this temporal contiguity (whether felt or remembered) to be non-permanent, since our lives are replete with examples of interruption and resumption of 1PP, such as in blows to the head, sleep cycles, and general anaesthesia.

This problem builds on critiques of persistent personal identity (Parfit, 1984). Regardless of whether a theorist argues for empty, open, or closed individualism in response to these challenges, they still need to account for the introspective phenomenology of self-persistence in a way that is consistent with the mechanisms they think generate, bind together, and place boundaries around conscious experiences.

At the simplest end, we might experience a moment bound together over at least a second or so—from the start of a musical note to its end, for instance. Does this correspond to a single bounded 4D entity, operating within a constrained spatio-temporal space? Or is it a spatially identically 3D space that persists over time without changing? Or is it spatially varying 3D spaces? If the latter, what about them joins them over time without extending before/beyond the desired period? Can we apply the same account for binding over a single second to binding over longer perceived persistence, such as a single waking day or a lifetime?

## 4. Topological segmentation of EM fields as a resolution direction

This section begins by explaining how field topology creates ontologically hard boundaries in principle. We then explain how particular topologies have the potential to meet three desiderata for any ToC: downward causation as a unit, no strong emergence, and frame invariance. We close by describing how field topology can be combined with an EM account of 1PP to account in principle for the five problems described in section “3. Precise statement of five specific boundary problems.”

### 4.1. How field topology can create hard boundaries

Field topology refers to the geometric properties of an EM field object that are preserved under continuous transformations, such as stretching, bending, or twisting (e.g., Rañada, 1989). In an EM context, topology can be used to describe the patterns of field lines or equipotential surfaces that define the distribution of the field. EM fields are associated with the movement of charged particles, such as occurs in abundance and diversity in the human brain (e.g., with many for every single neuron), giving rise to correspondingly complex and ever-changing field topology.

Within the complex topology of EM fields produced by the brain, we can consider what patterns might emerge with different levels of stability and at different spatial scales, from the sub-neuron level through to potential brain-wide fields (see section “5. Conclusion” for further discussion of spatial scales). One type of stability emerges when field lines occur in closed loops, potentially enclosing an EM field which itself might have complex patterns and vortices within it. As Wolski (2011) explains, lines of magnetic flux almost always occur in closed loops, whereas lines of electric field may occur in closed loops, but not necessarily.

Closed EM structures with certain durations can be understood as enclosing electromagnetic space so as to temporarily prevent the transit of energy with that same EM spectral range outside of the space.^6^ For its duration, there is an ontologically closed space for the relevant phenomena in that spectral range, in the sense that the information encoded in that field cannot exchange information externally on the same wavelength. The charged particles nonetheless continue to operate, subject to downward causation from the field, and as the closed structure collapses, causal interactions and information exchange with surrounding entities can continue.

One analogy is via the twisting of balloons to create knots or pinch points, such that it is possible to have complex inside/outside dynamics. Irvine and Bouwmeester (2008) employed such principles to identify solutions to Maxwell’s EM equations based on Hopf fibration (see Figures 1–3 in their paper). These solutions lead to various closed loop patterns, including knotted beams of light, and potential applications in fluid dynamics, plasma confinement, and particle trapping. An alternative mechanism generating closed EM activity is total internal reflection and the family of topologically stable solutions discussed by Fedchenko et al. (2022).

The construction of closed EM field loops in the form of light knots, which persist until some EM disturbance, has continued in recent years, with further work on isolated optical vortex knots (Dennis et al., 2010), identified knots in quantum field theory (Hall et al., 2016), and technologies for manipulating increasingly tunable optical vortices (Shen et al., 2019).

These applications being found in physics, information processing, and communication, as well as the complexity of potential EM field topologies at different spatiotemporal scales of the brain, are sufficient to motivate at a conceptual level the potential presence of topologically segmented, temporarily ontologically closed 4D pockets generated by the brain’s EM activity.

### 4.2. Potential for frame-invariant topologies that work as a unit to exert downward causation

#### 4.2.1. Frame invariance and desirability for consciousness-generating mechanisms

By framing boundaries in terms of topological features, we gain an important benefit: Lorentz invariance. Subject to various uncertainties and inconsistencies yet to be resolved (e.g., Melia, 2022; Frankel, 2023), modern physics accepts that relative speed and mass distort spacetime. Under special relativity, simultaneity is also relative to the reference frame. To the extent proposed consciousness generating mechanisms rely on synchronicity or in-phase frequencies at different locations (necessary if they are to resolve the binding problem between those locations, for instance), those consciousness would not be bound together from the perspective of anyone in any reference frame moving relative to the first.

If exact synchronicity or exact in-phase resonance is required, then even walking relative to someone else would be enough to disrupt it. If inexact synchronicity were sufficient, we should ask what threshold mechanism generates a qualitative distinction from along a spectrum of inexactitude or at least what mechanism generates the illusion of qualitative differences between the having of 1PP and not. One candidate threshold is the limiting temporal resolution of perception in that particular system, at the cost of allowing different thresholds in different systems or at different times. Alternatively, where inexact synchrony is enabled by design in the binding mechanism, similar thresholds of inexactness may be sufficient at spatiotemporal scales where any special relativity effects can be tolerated within the resulting fuzzy boundary definitions. For instance, physical objects can resonate with each other at similar but not identical frequencies; such coupled systems might then gradually exchange energy until they are exactly in sync (phase-locking). Hunt (2020) suggests several possible synchrony indexes that calculate synchrony as a continuum rather than all or nothing. Please see Hunt (2011) footnote 28 for a discussion of other reasons why special relativity may not be a barrier to synchronisation solutions.

Frame invariance may not be strictly necessary for consciousness, but it is likely to be desirable. For instance, if inexact thresholds do not apply, a lack of frame invariance suggests an unexpected proof of solipsism. Unless someone is exactly in my reference frame – hard to achieve, given the difficulty in coordinating small movements between bodies–then the only consciousness that binds together from my perspective is my own. Of course, everyone else is in the same camp, so their own 1PP may be consistent internally. Unfortunately, we can extend this argument to micromovements and changes within the body as well. If truly exact synchronicity is required, arguably there can be no binding within a brain either, at least not between a large number of points, as many are moving at very slightly different speeds to each other. For these reasons, if we wish to preserve special relativity (and good luck to anyone looking to replace it with something better), frame invariance is an attractive feature for consciousness generating mechanisms.

In the case of relativity, a particular type of frame invariance is necessary: Lorentz invariance (e.g., Ehlers and Lämmerzahl, 2006). This is sustained in the topological features of fields. Even though the specific timing of events and spatial distances may stretch and shift based on frames of references, topological features like interconnected areas, gaps, the aggregate of vortices and antivortices, knot structures, Euler characteristic, and boundaries remain consistent up to certain isomorphisms, as does the sequence of cause and effect. Provided it is these features that matter for the experience of 1PP, then a 1PP can be consistently generated within a brain despite micromovements and different internal frames of references and would still have the features necessary to identify as a 1PP for any external observer no matter their frame of reference.

#### 4.2.2. Desirability of non-epiphenomenalism

A further desideratum for consciousness-generating mechanisms is non-epiphenomenalism. We note this as a potential challenge for IIT, since the local maximum of phi is a denotive feature of a given subsystem that does not change what that subsystem does. For instance, the subsystem’s probability transition matrix can remain the same even if some other subsystem at a later stage ends up having higher phi and “takes over” the 1PP in the system.

Epiphenomenalism, in this context, is not the argument that phenomenal consciousness is entirely invisible to the world—if so, we could not be talking about it or experiencing it as an illusion or otherwise (see the meta-problem discussed in Chalmers, 2020; for possible defences of stronger epiphenomenalism see Robinson, 2019). Rather it is the weaker claim that the 1PP experience is a by-product of particular physical processes in the human system that does not directly causally interact with those particular processes. Classic examples are Huxley (1874) steam whistle on a train. The train’s motion causes the sound, which has a real physical existence and can influence other things (it can be heard and talked about), but does not influence the train’s motion, being what causes the sound. A stronger example might be an object’s shadow, since the whistle’s sound may have some trivial influence on the train’s motion, e.g., as it dissipates energy.

Non-epiphenomenalism is valuable for at least one of two reasons, depending on your philosophical position. If consciousness has no direct causal effect on the systems producing it, then it would be an extraordinary coincidence that natural selection appears to have universally selected for it in human organisms, and likely many other complex organisms as well. Extraordinary coincidences do happen, but mechanisms that explain why they were more likely (or indeed necessary) gain some plausibility as a result. Secondly, for some theorists, the combination of system-level causation with a 1PP provides a route to rescue the sensation of free will in an otherwise deterministic universe (e.g., McFadden, 2006). Weak emergence provides one channel to deliver non-epiphenomenalism.

#### 4.2.3. Weak emergence as a route for non-epiphenomenalism

Different topologies of EM fields have been shown to operate as a unified whole that has weakly emergent, downward causation on the types of activities that happen and are possible in their neighbourhood. McFadden (2013) provides a discussion of non-epiphenomenality in EM fields, with research continuing to make progress in recent years. For instance, Pinotsis et al. (2023) draw together work on ephaptic coupling: showing how electric fields sculpt neural activity in the context of brain infrastructure, potentially tuning it to process information more efficiently, as well as influencing memory formation (Pinotsis and Miller, 2023). In other words, EM fields are not merely a side-effect of electrical activity in the brain, but in fact influence the activity of individual neurons and their parts.

To provide some illustrative references for this paper of how field topology in particular has been found to have downward causality: experimentally observed differences in resonances when light transits a Möbius strip topology compared to an ordinary ring (Wang et al., 2023), the proposed topological dynamics of skyrmion bundles (Tang et al., 2021), and perhaps most dramatically the release of twisted magnetic field structures in the sun causing coronal mass ejections (NOAA, n.d.).

A brief definition of weak emergence is in order, where causal influence rather than ontological fundamentality is sufficient to support non-epiphenomenalism. In this paper, weakly emergent causality is where a structure influences the behaviour of its constituent parts, perhaps by constraining the space of actions available to individual parts. However, that structure and its properties are fully defined, albeit potentially incompressibly, by the (local) interactions of those parts in the given environment. For a fuller discussion and opposing positions, please see O’Connor (2021).

Two notes are worth appending to this definition. While all interactions are local, they might only exist the way they do with the full structure in place, as each local interaction is itself constrained/shaped by its neighbouring interactions, recursively through to the whole structure. Secondly, while the outcomes are fully defined (or vary only by some irreducible randomness), they may still be unknowable or incomputable in practice to an observer, e.g., uncertainty principles, non-linear macrosystem dynamics, and chaos theory are all consistent with this weak emergence. By contrast, strongly emergent causality entails some inherent nature of the system that cannot be predicted or explained by the lower-level components alone, even with perfect knowledge, in a way that goes beyond these computability or observability caveats.

One toy example is provided as an intuition pump. Traffic congestion is a dynamic property emerging weakly from entirely local interactions among the more fundamental unit of vehicles operating in a given environment. Once that property has emerged, a fundamental unit’s space of operations is constrained by the property relative to what it has if isolated (e.g., the car can no longer drive over 10 mph if that is the congested speed). Only “local interactions” are ontologically real (in this toy universe), but their dynamics still matter in a way that is not solely perspective dependent. Emergent structures can, for instance, result in features that can “block passage”: exactly what is needed to solve the boundary problem.

#### 4.2.4. Weak emergence in the brain

The examples of weak emergence from fields span a sufficiently wide range of endeavours that we might reasonably expect to find them in the human brain if properly analysed. Such downward causation helps to explain why the mechanism might be visible to evolutionary processes. If it is visible and has benefits in some circumstances, then we would expect it to have been co-opted by natural selection on at least some evolutionary pathways. Combined with the anthropic principle (we can only comment on its presence, since in its absence we would be unable to comment on it), it is no longer surprising that individual humans have a 1PP.

The relatively weak downward causation from the field, relative to say the computational behaviour of neuronal interactions, can be construed perhaps surprisingly as an asset of the theory. As is widely known in neuropsychology, the brain does an enormous amount of processing at the subconscious level. Our conscious experience is often of making relatively few, relatively focussed decisions. Downward causation from a weakly emergent structure may only make a difference in a few circumstances, aligning with this experience. For instance, when complex systems are close to criticality and unable to predict themselves, the occurrence of one route over another may be deliverable via a small nudge of downward causation from a field integrating information surfaced to it at the endpoints of various complex computational modules.

This account of decision making is similar to a central coordinator or global workspace function, with the unified 1PP providing the glue that binds it all together. Where that field is a 1PP, it is understandable that this perspective experiences a sensation of choosing and, depending on your philosophical position, exerts its influence over the outcome based on its assessments, corresponding to what could be called a sensation of free will.

### 4.3. Applying topology in an EM-field ToC context to address the boundary problems

Field topology may be a useful tool for all EM-field theories to use, as discussed further in the conclusion. In this section, we present a conceptual account of how we might address the boundary problems where a 1PP arises in any 4D-topological pocket, i.e., an EM field pattern which provides hard boundaries around a specific object in spacetime. We will equate a 1PP ontologically and axiomatically with fields shaped into such bounded pockets. Other theorists may successfully draw on the topology principles to resolve the boundary problems but relate the underlying 1PP to other features, such as the field’s energy (Jones, 2010) or its information content (McFadden, 2020).

#### 4.3.1. Addressing the first problem

The first problem is resolved in a straightforward fashion given section “4.1. How field topology can create hard boundaries,” asserting that the ontological boundaries of the pocket are sufficient to account for the hard boundaries we typically experience. Binding within the topological pocket is explained in the traditional EM field sense, noting that fields are unified by default. The complexity of the contents of unified experience, i.e., often containing multiple shapes or features, is explained because the field contains all the information of the EM activity that gives rise to it, including computational insights and assessments from relevant diverse brain modules operating via a neuronal architecture. Local perceptual binding, such as binding the colour green to the shape of a tennis ball, might also be supported topologically, for instance with the relevant features bound along a certain axis (e.g., a 2D vortex) but not fully bound when viewed from a 4D perspective, else it would itself satisfy the conditions necessary to provide a 1PP itself. Such topology may itself be the EM outcome of underlying neuron-based computations to analyse perceived features, as popular in computational neuroscience, computer vision, and the earlier discussion of predictive processing.

We acknowledge that the presented EM fields solution to the boundary problem is conditional on their solution to the binding problem being effective (see citations in section “2. Literature review of the boundary problem”) and accurate to the human experience, noting that other explanations for phenomenal binding have been proposed. This conditionality could turn on future insights from physics and metaphysics, depending on whether the particular phenomenon that creates binding must be ontologically fundamental or can be an emergent structure. Some researchers from section 2. “Literature review of the boundary problem” may accept the latter (e.g., resonance requires a substrate), whereas others may assert the former (e.g., quantum entanglement). However, with no strong consensus on what is ontologically fundamental, progress is limited. Traditional views of particles (in the Standard Model) and space-time plus mass-energy (in general relativity) are known to be incomplete and are under challenge, with contenders arguing that the base layer of reality is variously fields (QFT, e.g., Peskin, 2018), information (Wheeler; see Plastino, 2004), multi-dimensional strings (Greene, 2000), mathematics (Tegmark, 2014), mental objects (Kastrup, 2019) or conscious agents (Hoffman and Prakash, 2014), among others.

Rather than one fundamental object and its interactions, it is also possible that multiple objects co-exist at the base layer or they interact over more dimensions than we can sense [e.g., Ney’s (2021) discussion of wave function realism as a “local” resolution to apparent quantum non-locality]. There is even disagreement over whether the discipline of physics is capable of probing fundamental ontologies, perhaps being restricted to the results of relationships between whatever is fundamental (Russell, 1927; Jones, 2010; Goff, 2020). Through this lens, asserting what is ontologically fundamental may be unproveable in traditional scientific experiments but alternatives can still be debated rationally, considering which candidate axioms best satisfy a useful set of specified properties.

#### 4.3.2. Addressing the second and third problems

The second and third problems are resolved by accepting all well-bounded 4D topological pockets to have their own 1PP, potentially of a very rudimentary and short-lasting nature. Depending on the mechanisms involved, there may be dozens or billions of these in any one system, both smaller than and larger than the meso-level humans typically experience and discuss with each other. Our inability to identify them is ultimately an empirical question of analysing and measuring field topology, not a firm epistemological boundary. Nonetheless, we would not take the identification of very many such pockets to be a fundamental challenge to the theory.

Rosenberg (2004, s4.8) worries that such an approach is “panpsychism run wild,” arguing that an explanation “that promiscuous is not illuminating.” We politely disagree. The promiscuity of such 1PPs is no more counter-intuitive, we suggest, than the multitude of smaller objects we are already made up of. A total of 30 trillion cells in the human body, some seven octillion atoms (10^27), and an absurdity of quarks and gluons popping in and out of existence in the tiniest of seconds. The universe is home to an estimated 10^25 planets orbiting stars and perhaps 10^80 atoms. Our human-level intuition already glosses over these unfathomably large numbers because we have chosen to accept them; the same is possible of the mind-dust corresponding to topological pockets. To the extent that ants have a rudimentary consciousness, there are an estimated 20 quadrillion on earth. That certain phenomena exist in larger numbers than we normally observe is no reason to deny them.

If there are indeed a possibly large number of 1PPs nested within and beyond us, the second and third questions also ask why our 1PP remains relatively stable at the meso-level. Our answer to this simultaneously addresses the fifth problem by reference to the problem of identity at different scales.

#### 4.3.3. Addressing the fifth problem

We consider three temporal scales to illustrate the potential mechanisms at work: micro (sub-second), single experience (e.g., several seconds, perhaps several minutes or hours in some cases), and lifetime (e.g., years or decades).

Without prejudicing future empirical investigation, we will consider a 4D topological pocket object that spans a modest proportion of the human brain in spatial terms (perhaps several centimetres) and short in temporal duration (perhaps a few milliseconds). The pocket is naturally defined over the fourth dimension (time)—indeed it may fail to have hard boundaries without the topology along its temporal dimension. Thus there is intrinsic temporal depth at the micro-level for the 1PP. However, the 1PP corresponding to that pocket only exists for that short duration. As the underlying EM activity changes (different neurons fire etc.), the field topology changes and a new 4D pocket emerges, which similarly satisfies the hard boundary conditions described in section “4.1. How field topology can create hard boundaries.” At least some such pockets emerge predictably and consistently, since there is part of the brain optimised through evolution to generate them, recruiting the power of such fields to enhance information processing. This new 4D pocket is a new 1PP. Each one exists for a short period of time and almost certainly less than a few seconds in normal human experience.

What causes these ontologically distinct 4D pockets to link together over time at the scale of short experiences, e.g., parsing a sentence or enjoying a song? This is the key step for explaining the uniformity of our meso-level experience. Of all the well-bounded 4D topological pockets that might exist in the brain, we suggest that only one of them bounds a field that encloses (and hence integrates) EM activity emerging from the brain’s immediate memory modules.

Various other modules may also be surfacing information that is bound into the pocket, whether those are making sense of our perceptions to construct the indirect realist world we experience, considering actions and decisions against some set of goals, or various other mental functions we experience. In the context of continually jostling topologies, we can imagine these different modules producing EM fields that “compete” to contribute information to the well-bounded 4D field that is integrated with the relevant memory modules, helping to account for the phenomenology of multiple inputs competing for our attention that inspires advocates of global workspace theories.

The necessary step is that the topological pocket of interest integrates information from sequential instances in the recent and immediate past. It is the immediate memory module whose EM activity surfaces that information into each sequential 4D pocket. Unless that module is designed to surface similar outputs to different parts of the brain (a potentially evolutionarily costly redundancy), the requirement that any one piece of EM activity can only be enclosed by a single closed pocket at any one time provides the uniqueness constraint. The time durations from memory overlap from pocket to pocket, creating a more prolonged sense of time or a “pseudo time-arrow” than would be present in any single 4D pocket that does not enclose the memory input. Other 4D pockets, however many might exist, evaporate almost as soon as they begin: mind-dust with no sense of persistence.^7^ In setting out a conceptual direction for resolution at this stage, we are not specifying which specific part(s) of the brain might be necessary for this immediate memory function or whether they correspond to specific theories of memory/awareness that remain active areas of study.

The key thing for our experienced persistence over time is that link to memory—the constant and repeated referencing of memory by different, consecutive, internally bound 4D pockets is what creates the sense of persistence. Persistent identity over a lifetime, weak as it is, is then generated by references to longer term memories and senses of self. Such an account is consistent with observations that our self can change over time, especially over decades, and that our short-term sense of self can be disrupted by memory disorders or certain chemicals.

#### 4.3.4. Addressing the fourth problem

The merging of two 1PPs into a single 1PP is only a relevant question for a single 4D pocket in any case, existing for a very short period of time. This may be possible in principle but extremely difficult, since any attempt to bring the necessary modules close enough would likely destroy the physical mechanisms that generate a pocket with the right hard boundaries to enclose a 1PP with any temporal persistence (even microseconds). The merged field may also be sufficiently different to the two original 1PPs that it is more meaningful to talk of a new 1PP than a merger.

Perhaps certain cases of conjoined twins and shared awareness are cases of such joining happening and undoing itself at different points in time with different topologies. However, we suggest it is more likely that such cases reflect information being shared between 1PPs, even jointly and simultaneously surfaced by the same brain mechanism. From this perspective, privacy is more about consenting to communication, rather than ontological separation, and is likely more a technological issue than a metaphysical one.

## 5. Conclusion

This paper has re-introduced and refined the boundary problem for theories of consciousness, as puzzled Schrödinger (1951) and first framed in detail by Rosenberg (1998). Inspired by Rosenberg’s account, we have specified five problems which can be considered siblings to the famous binding problem, in that any ToC which provides solutions to the binding problem must also provide an account for our five problems. To a first approximation: if mechanisms are proposed to bind phenomenal experiences into a unity, what is it that stops the mechanism from expanding? What puts boundaries around those unified experiences and why do the boundaries appear as they do?

Our literature reviews found very little scholarly discussion of the boundary problem, at least as compared to the binding problem. Some promising avenues to resolving aspects of the boundary problem can be found in the phase transitions motivated in some resonance, quantum field, and EM field ToCs. However, none of these ToCs yet provide a full account against all five problems we specify. Specifically for field theories, we introduce a physical feature that may be particularly useful for tackling the problems: topological segmentation. Without prejudicing the possibility of other accounts, we provide one conceptual account by which 4-dimensional topological pockets could address all five problems while meeting other ToC desiderata: non-epiphenomenalism, no strong emergence, and frame invariance.

Our purpose in this paper is not to advocate for any one EM field ToC. The topological segmentation we describe can operate, in principle, at different spatiotemporal scales. For instance, referencing the citations in section “2. Literature review of the boundary problem”: Shani and Keppler might motivate it to create relevant boundaries at the tiny scale of ZPF attractors in SED quantum fields. Jones might use it for the highly localised fields along ion channels where he identifies the seat of consciousness. Ward and Guevara might examine the topology of EM fields produced around the thalamus to find the right boundaries, McFadden might find them at the whole brain level, or Bond might even find them beyond and between brains.

An indirect contribution of this paper is to provide a further tool for differentiating between these EM field theories. If we accept field topology as the solution to the boundary problem in human consciousness, then the exercise of testing whether boundaries exist becomes a mathematical and empirical exercise that might proceed in three stages. In the first stage, the relevant part of the brain should first be modelled to capture as much relevant EM-field producing activity as possible, building on the high-level EM field mapping of the brain by Singh et al. (2019). This relies on a combination of imaging, dissecting, and computational exercises, similar in spirit to the Human Brain Project for mapping our neurons,^8^ as already successful for the fruit fly larva connectome (Winding et al., 2023).

Secondly, the topology of the resulting field must be analysed to identify where there are closed loops (or alternative topological features that draw boundaries in the fields) that create the relevant hard boundaries at the theorised scales.

Finally, and subject to safe and ethical design, targetted EM pulses might be used to disrupt the identified topology, testing whether subjects’ experience of consciousness varies in the required manner. If the boundary of consciousness itself is disrupted by such efforts, then we would expect the 1PP to collapse temporarily before returning (similar perhaps to deep sleep or cessations in meditation), rather than merely altering the content of what the 1PP is conscious as part of a continuously conscious experience.

We predict that such exercises will only identify topologies at a broadly consistent spatial scale for the presence/absence of 1PP, although impacts elsewhere in the brain might affect what that 1PP is conscious of. Whether this scale turns out to be micrometres, millimetres, centimetres, or decimetres will help narrow down to the correct EM field theory. The success or failure in identifying such topologically hard boundaries will also inform any competing introspective intuitions about the hardness or fuzziness of the phenomenological boundary discussed in section “3.1. The hard boundary problem.”

As a practical first step, the authors are developing a technical companion paper to provide a mathematical and heuristic description of the conceptual model outlined here. Such a paper will discuss the kinds of closed boundaries that might plausibly occur within common, simplified brain anatomy and how topological pockets inter-relate. In doing so, we are inspired by mathematical work in topology and phenomenology by such scholars as Baudot (2018), Prentner (2019), and Mason (2021). Such work can help find topologies that are likely to exist given the EM activity in the brain, that create hard boundaries, that have sufficient complexity in a 4D space to reflect our multi-featured macrophenomenology, and that have irreducible computational benefits. This research direction is supported also by the growing empirical research base around wave dynamics in human brain geometry (Pang et al., 2023), cytoelectric coupling in the brain (Pinotsis et al., 2023), neural field analysis (e.g., Robinson et al., 2016), and the broader literature on cross-frequency coupling and phase-amplitude coupling. The full three stage research vision is a major scientific task, but should be accessible to the same human ambition which landed a robot on Mars for US$ 1 bn,^9^ found the Higgs Boson in a machine with some US$ 10 bn of budget,^10^ or achieved the first full mapping of the human genome at an estimated total cost of US$ 0.5-1 bn.^11^ The prize is surely also no smaller: understanding the mechanisms that give rise to our first person perspective, in a way that explains why we experience it as unified and bounded at the scale we do.

## Data availability statement

The original contributions presented in this study are included in the article, further inquiries can be directed to the corresponding authors.

## Author contributions

AG-E came up with topological segmentation as a potential solution to the boundary problem, as well as core arguments around non-epiphenomenalism, and applications of topological segmentation in current research. CP conducted the literature review, developed the five boundary problems, and constructed the illustrative EM field account that could address all five problems. Both authors contributed equally to the manuscript, writing the manuscript and to testing, and refining the arguments throughout.

## Footnotes

1 Consistent in the sense of accepting the self-reported sensations of such experiences, rather than necessarily the ontology that some might adopt to explain them, being topics for separate papers.

2 In general, we use “brain” as a concise, common synecdoche for a central nervous system embodied in a functioning human system, acknowledge imprecision and uncertainty around exactly which components are essential.

3 Chalmers (2016) does not use the phrase “binding problem” as part of the family of combination problem issues, but other researchers have used language that connects to the subject combination problem, e.g., Garson, 2001 account of phenomenal binding.

4 The other two are: Hunt (2011) which equates the boundary problem to James (1895) account of the combination problem (which does not address Rosenberg’s full concerns). In Hunt (2011) later work, cited here, the concerns are addressed more directly. The second is entirely out of scope, concerning the interpenetration of consciousness and media technologies.

5 Various theories provide accounts for feature binding for specific subcontents of conscious experience, without necessarily seeking to explain the overall unity of phenomenal conscious experience. For instance, in the Seth and Bayne (2022) account of predictive processing theories, when the brain uses learned or inferred patterns to predict what a partly perceived object should be, the unity of that informational pattern naturally links features together. Even if such computational approaches prove correct for feature binding, a separate mechanism would likely be required for overall 1PP unity, given the challenges highlighted in section “2.1. Rosenberg’s formulation of the boundary problem.”

6 Some related phenomena might trivially transit this boundary (e.g., sound waves, x-rays) and others (e.g., WIMPs) could transit any other currently conceivable boundary relevant to everyday human experience. The key requirement is that the relevant phenomena that construct the closed field, i.e., the same EM field, do not transit the boundary.

7 Whether you consider the phenomenological persistence we describe between pockets as illusory in some relevant sense or real is perhaps a matter of personal aesthetics and in any case a subject for a separate paper.

8
www.humanbrainproject.eu

9
https://www.planetary.org/space-policy/cost-of-the-mars-exploration-rovers

10
https://en.wikipedia.org/wiki/Large_Hadron_Collider

11
https://www.genome.gov/about-genomics/fact-sheets/Sequencing-Human-Genome-cost
