# Corrigendum: Don't forget the boundary problem! How EM field topology can address the overlooked cousin to the binding problem for consciousness

**DOI:** 10.3389/fnhum.2024.1373466

**Published:** 2024-02-01

**Authors:** Andrés Gómez-Emilsson, Chris Percy

**Affiliations:** ^1^Qualia Research Institute, San Francisco, CA, United States; ^2^College of Arts, Humanities and Education, University of Derby, Derby, United Kingdom

**Keywords:** consciousness, binding problem, combination problem, boundary problem, electromagnetic fields

In the published article, there was an error in the Abstract. An author's name was spelt incorrectly.

This sentence previously stated:

“By comparison with the phenomenal binding problem, the boundary problem has received very little scholarly attention since first framed in detail by Rosengard in 1998, despite discussion by Chalmers in his widely cited 2016 work on the combination problem.”

The corrected sentence appears below:

“By comparison with the phenomenal binding problem, the boundary problem has received very little scholarly attention since first framed in detail by Rosenberg in 1998, despite discussion by Chalmers in his widely cited 2016 work on the combination problem.”

The authors apologize for this error and state that this does not change the scientific conclusions of the article in any way. The original article has been updated.

